# Learning from the Montreal Protocol to improve the global governance of antimicrobial resistance

**DOI:** 10.1136/bmjgh-2024-015690

**Published:** 2024-10-03

**Authors:** Tina Nanyangwe-Moyo, Gabriel C Fezza, Susan Rogers Van Katwyk, Steven J Hoffman, Arne Ruckert, Samuel Orubu, Mathieu JP Poirier

**Affiliations:** 1 Global Strategy Lab, York University, Toronto, Ontario, Canada; 2 Osgoode Hall Law School, York University, Toronto, Ontario, Canada; 3 School of Global Health, York University, Toronto, Ontario, Canada

**Keywords:** Health policy, Environmental health, Global Health, Public Health

## Abstract

The Montreal Protocol has played a critical role in promoting global collective action to phase out the use of ozone-depleting substances, ultimately preventing millions of cases of skin cancer, cataracts and other health issues related to ultraviolet radiation exposure. This success entails transferable lessons for coordinated action required to improve the global governance of other challenges. Like ozone depletion, antimicrobial resistance (AMR) is a challenge of the global commons, requiring coordinated actions across human, animal and environmental sectors. We identify equity, flexibility and accountability as three core governance principles that underlie the success of the protocol and employ the 3-i framework to understand how interests, ideas and institutions contributed to the protocol’s success. Equity-promoting strategies consisted of an inclusive negotiation process, supporting developing countries with multilateral funding and a progressive compliance model. Flexibility was built into the protocol through the development of country-specific strategies, reorienting incentive structures for industry and facilitating regular amendments in response to emerging scientific evidence. Accountability was promoted by mobilising public advocacy, establishing targets and enforcement mechanisms and conducting independent scientific and technical assessments. Applying our proposed principles presents an opportunity to improve the global governance of AMR. Finally, we acknowledge limitations to our analysis, including our focus on a single environmental treaty, significantly greater funding requirements and multifacetted stakeholder involvement in the case of AMR, differing market and incentives structures in antibiotic development and distribution, and ethical concerns with using trade restrictions as a policy tool.

**SUMMARY BOX**

- The rise of antimicrobial resistance (AMR) is one of the most pressing contemporary challenges for human and environmental health and will require coordinated global efforts across human, animal and environmental sectors.
- The Montreal Protocol provides an example of successful global collective action to address a challenge of the global commons with important implications for the global governance of AMR.
- We identify nine principles from the design to implementation of the Montreal Protocol that can be applied to ongoing negotiations of an international agreement to govern AMR.
- Overcoming AMR challenges such as uneven antimicrobial stewardship and catalysing investment in the discovery of new antimicrobials will require sustainable multilateral funding, multisectoral coordination and strong global advocacy.

## Introduction

 The Montreal Protocol, a legally binding treaty to tackle ozone depletion that went into force on 1 January 1989, is widely recognised as a model of success in addressing a global collective action problem. A little over a decade after scientists first suggested that chlorofluorocarbons (CFCs) would deplete the Earth’s ozone layer, a startling drop in stratospheric ozone concentration was discovered in 1985 over Antarctica.^1 2^ The ozone layer absorbs most of the sun’s ultraviolet radiation, preventing damage to animals, plants and microbes, and decreasing risks of skin cancers and cataracts among humans.^3^

**Figure 1:**
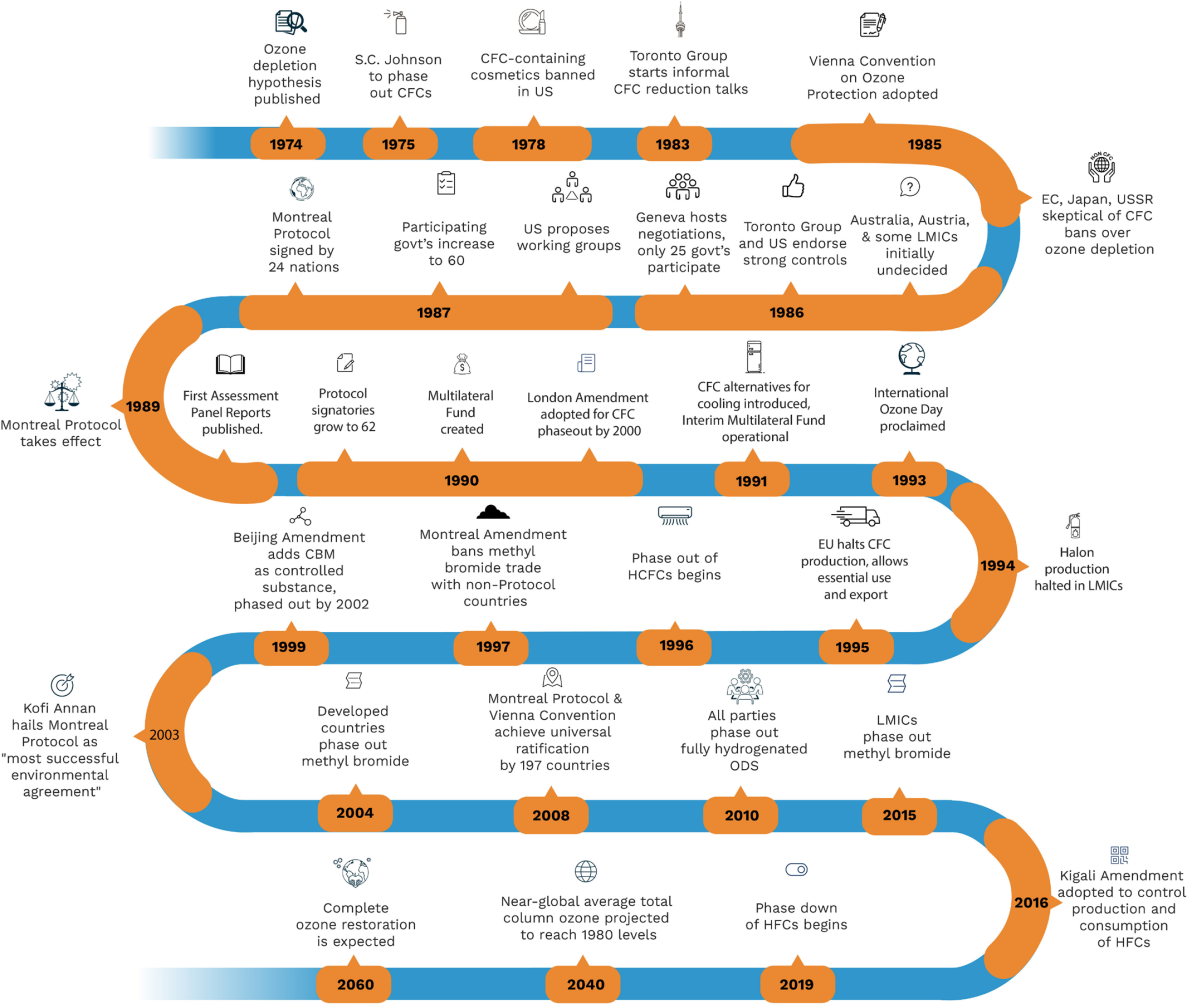
Notable events in the negotiation and implementation of the Montreal Protocol. CBM, chlorobromomethane; CFCs, chlorofluorocarbons; EC, European community; HFCs, hydrofluorocarbons; LMICs, low-income and middle-income countries; ODS, ozone layer-depleting substances.

Within 2 years of this discovery, the protocol was negotiated and signed to phase out ozone layer-depleting substances (ODS), including CFCs.^4^ This speed was remarkable because this new global challenge posed a threat to refrigeration, air conditioning and aerosol propellant industries worth more than US$350 billion in current US$ in the USA alone,^5^ and the United Nations had only recently taken up the governance of environmental concerns in a world divided by the Cold War. Despite some uncertainty on the role of the protocol in accelerating global reductions of ODS emissions,^6^ it was instrumental in galvanising an emerging scientific consensus, which strengthened an international advocacy coalition to leverage new regulations introduced by the United States’ Environmental Protection Agency.^7 8^ Nearly 40 years later, the ozone layer is on a path to recovery, with restoration to 1980 levels expected by 2040 (figure 1).^9^

Antimicrobial resistance (AMR) is one of the most pressing contemporary challenges for human and environmental health, and—like the destruction of the ozone layer—will require a coordinated global effort.^6 10^ AMR is a natural process by which microbes become resistant to antimicrobial agents; however, the proliferation of drug-resistant infections around the world has been massively accelerated by the overuse and misuse of antimicrobials in both humans and animals.^7^ Although international efforts are increasingly mobilised through the UN General Assembly, the Global Leaders Group on AMR, and the Quadripartite Joint Secretariat, projections indicate that resistance to last-resort antibiotics could double by 2035.^8^ This daunting reality has led to repeated calls for an international legal instrument to rally global action to address rising AMR.^6 10^ As of July 2024, the most recent draft of the Pandemic Treaty lacks AMR-focused provisions, prompting suggestions to develop an AMR protocol of the Pandemic Treaty, among other potential options.^11^

In this analysis, we investigate lessons from the Montreal Protocol for ongoing and future efforts to manage the global antimicrobial commons. Previous studies examining the Montreal Protocol in the context of AMR have highlighted the importance of multilateral funding (MLF) mechanisms, fairness and flexibility to incentivise state participation and adherence.^11^,^13^ Our analysis updates this work amid a rapidly evolving political context and employs a 3-i policy framework that has been previously used to analyse the global policy response to COVID-19^14^ and global health diplomacy practices,^15^ but has not yet been used in the field of AMR governance.

We employ the 3-i framework to explore how interests, institutions and ideas were mobilised to achieve progress in restoring the Earth’s ozone layer.^16^ The 3-i framework assumes that policy developments are influenced by actors’ interests and ideas, as well as by institutional set-ups and looks for entry points to facilitate policy implementation.^17^ We used a snowball approach to search JSTOR, an extensive digital library with relevance to our research interests given its broad focus, for key terms such as: ‘Montreal Protocol’, ‘negotiation’, ‘compliance’, ‘amendment’, ‘ODS alternatives’, and ‘phaseout schedules’ to identify relevant information. Our search yielded more than 250 resources, which were then assessed for relevance using an internally developed screening tool to include articles that had relevance to at least one of the following aspects: treaty negotiation, treaty implementation or strategies employed to resolve uncertainties and barriers to the agreement obligations.

Data were organised into the three inductively emerging themes of *equity*, *flexibility* and *accountability*, and under each category, we identified principles aligning with interests, ideas and institutions (*see[Table T1]* table 1). Through this process, we identified nine governance principles across three themes that catalysed the protocol’s success and drew transferable lessons for improving the global governance of AMR.

**Table 1 T1:** Lessons from the Montreal Protocol and implications for AMR

	Lessons from the Montreal Protocol	Implications for AMR
Equity		
Inclusive negotiations (Interests)	Conflicting state-party interests were reconciled by promoting early and active participation of developing countries in treaty negotiation.	Need for inclusive decision-making processes that support the needs of LMICs and diverse country contexts. Mobilise adequate and sustainable funding to support AMR containment measures and discovery of new antimicrobials. Develop an adaptive progressive phaseout of antimicrobial use in food-producing animals contingent on country-level economic and nutritional status.
Multilateral funding (Institutions)	Adequate and sustained funding was mobilised to support research and development of ODS alternatives.	
Progressive compliance model (Ideas)	Article 5 allowed select countries a 10-year delay in meeting targets with continued access to ODS as alternatives scaled up.	
Flexibility		
Country-specific strategies (Interests)	Flexible and adaptive approaches inspired country-specific strategies to implement ODS control measures in diverse contexts.	Allow for common but differentiated responsibilities for achieving AMR targets and compliance measures. Establish industry incentives and regulations to support development and stewardship of new antimicrobials. Adopt a framework that allows iterative changes to NAPs and international law based on emerging scientific evidence.
Reorienting incentive structures (Institutions)	Concessions and tailored incentive packages for industries won institutional buy-in to reprioritise investments to develop ODS alternatives.	
Regular amendments (Ideas)	Adaptable treaty provisions allowed new findings from scientific, environmental and technological assessments to be adopted into action.	
Accountability		
Public advocacy (Interests)	Mobilising public support from civil society and academia motivated government action.	Link academia, patient advocacy groups and others to build momentum for government action. Set global goals and country-specific targets for reducing AMR and use trade pressures to incentivise participation. Expedite the establishment of an Independent Panel on Evidence for Action Against AMR, proposed in 2019 by the Interagency Coordination Group.
Targets and enforcement mechanisms (Institutions)	Strict reduction schedules for ODS paired with trade provisions incentivised ratification by other countries.	
Independent external assessments (Ideas)	Established three assessment panels as independent monitoring mechanisms under Article 6.	

Themes of equity, flexibility and accountability are further divided by interests, institutions and ideas.

AMR, antimicrobial resistance; AMU, antimicrobial use; LMICs, low-income and middle-income countries; NAPs, national action plans; ODS, ozone-depleting substances.

## Governance principles and lessons for AMR

### Equity

Recognising structural imbalances in power, resources and capabilities, equity-promoting provisions in the protocol were key to facilitating cooperation and assistance between developed and developing nations and promoting shared responsibility in research, technology development and knowledge sharing.^18^

**Inclusive negotiations (interests)**: Reconciling conflicting interests of countries at the negotiating table was crucial for the protocol’s success. Developing countries that were reliant on ODS with limited access to alternatives, such as China, India and Mexico would demand a different approach to phasing out their use than countries with high reliance but with access to emerging alternatives, such as Germany, Japan and the USA.^18 19^ The transnational harmful impacts of ozone depletion and benefits of collective action to cut ODS emissions incentivised broad participation across divisions of geopolitical alliances and development status.^20^ Representatives from countries such as Brazil, Egypt, Kenya and Venezuela had significant roles in representing the perspectives of developing countries, despite not initially included in the ‘Toronto Group’, which first took up the movement to regulate ODS (see figure 1).^21^ These inclusive negotiations influenced the drafting of several equity-focused provisions, like Article 5 adapting targets and incentives to the ‘special situations of developing countries’.^4^

**Multilateral funding (institutions)**: The cost of developing new technologies to phase out ODS-emitting products and the lack of access to alternatives were significant concerns for developing countries. Even if political commitments for international assistance could be secured to support these countries, the institutions needed to finance and allocate funds to initiatives around the world did not yet exist. In response, the MLF was created to fund and facilitate research, development and knowledge transfer of ODS alternatives.^22^ The MLF has since supported over 8600 projects in industrial conversion, training and capacity building worth hundreds of millions of dollars across the world.^4^ This fund has been critical to supporting multilateral cooperation, country-level ODS reduction programmes and international monitoring and evaluation.^23^

**Progressive compliance model (ideas)**: Country-specific phaseout schedules were outlined in Article 3, taking annual production, imports, exports, consumption, emission levels and ozone-depleting potential for different ODS into account.^4^ However, ideas put forward during negotiations led to the adoption of differentiated phaseout schedules for developing countries. Recognising inequities in the capacity of some countries to meet time-bound targets, the protocol established a progressive compliance model accounting for imports and exports of ODS and development status. This allowed over 140 developing countries, or ‘Article 5 countries’, that consumed low levels of ODS (≤0.3 kg per capita) an additional 10 years before initiating reduction measures.^22^ Although these provisions delayed full implementation of the protocol, they eased transition to alternative substances for countries that faced barriers.

**Lessons for AMR**: AMR is a transnational challenge, with a rising burden that will heavily impact developing countries.^24^ Common, but differentiated, responsibility is necessary to protect and manage the global antimicrobial commons, with providing access to antimicrobials more urgent than establishing antimicrobial stewardship programmes for some developing countries. These countries face a double burden of access and use, where financial, fragmented regulatory standards and structural barriers impede consistent access to life-saving antimicrobials besides infrastructural challenges leading to overuse and misuse.^25 26^ As with ozone depletion, AMR will require inclusive decision-making processes, a progressive compliance model, as well as sustained commitment to funding obligations. Lastly, progressive compliance speaks to the importance of acknowledging different resource capacities, crucial for the implementation of National Action Plans (NAPs) and subsequent policy uptake.^27^

### Flexibility

A flexible approach to ODS control measures allowed for adjustments to be made to the protocol based on emerging scientific understanding and technological advances. While some countries developed ambitious plans to reduce ODS as part of an economic strategy, other countries needed significant reorientations in existing incentive structures.

**Country-specific strategies (interests)**: Negotiators recognised the diversity of economic and political interests at play, empowering countries to develop their own plans and incentive structures to facilitate the successful implementation of ODS control in diverse contexts. The Technology and Economic Assessment panel was created to convene industry experts to identify potential technologies and recommend industry efficiency standards to inform country plans.^28^ This flexibility in country-specific strategies led to the establishment of bilateral partnerships to promote access to emerging technologies. For example, ODS-emitting industries in Mexico and Thailand adopted more stringent controls than needed to match economic competition in developed countries.^29^ With technical assistance from Japan, industries in Thailand phased out ODS use 1 year ahead of their domestic schedules.^29^

**Reorienting incentive structures (institutions)**: Persuading companies to reprioritise investment, develop new technologies and switch production to ODS alternatives required institutional changes in both private and public sectors.^30^ To expedite commercialisation of non-proprietary technology, industries were offered incentives to mobilise their expertise and facilitate knowledge sharing.^22^ As demand for ODS substitutes grew, governments created demand for industry to act through initiatives like the US ‘golden carrot’ programme to commercialise superefficient refrigerators.^31^ By early 1990s, industry cooperatives sprung up, such as the Halons Alternatives Research Corporation (HARC). HARC led the development of hydrofluorocarbons (HFCs) as a transitional ODS substitute used in fire protection.^32^

**Regular amendments (ideas)**: The Montreal Protocol adapted to new ideas in the evolving landscape of ozone depletion, informed by new findings and proposals from scientific, environmental and technological assessments. Up-to-date atmospheric monitoring data allowed the protocol to have a dynamic legal framework through recurring meetings of countries every 4 years.^29^ Regulations to amend the protocol have added chemicals to the list of controlled substances and revised phase-down timelines based on the ability of countries to achieve set targets. For example, the 2016 Kigali amendment to reduce the use of HFCs by 85% by 2036 was based on contributions from both academic and state actors to implement proposed controls.^23^

**Lessons for AMR**: A global response to AMR will require multisectoral coordination and flexible governance systems, with unifying goals at the global level and nationally determined targets that are responsive to countries’ unique contexts and needs. Recognising the need for flexibility, the UN Quadripartite has embraced a strategy whereby countries establish their own NAPs to respond to local needs and capacities. There is a further need for flexibility in the global model for developing new classes of antibiotics to treat resistant strains, such as Gram-negative bacteria which causes around 70% of infections in intensive care units.^10 33^ Pull incentives for new drug development, like the Longitude Prize and the Trinity Challenge, are promising initiatives.^34 35^ Still, antimicrobial discovery and development face high costs, lengthy approval processes and effective stewardship measures, which discourages the needed private sector investment.^36^ Overcoming this dilemma will require a flexible approach to balance the need to stimulate innovation while balancing effective stewardship, regulation and equity.

### Accountability

Accountability mechanisms have compelled countries to meet their commitments and to regularly report on their progress. These mechanisms have been pivotal in tracking progress, driving compliance, ensuring global cooperation and maintaining credibility of the protocol since its adoption.

**Public advocacy (interests)**: Public mobilisation by scientists-turned advocates, environmental non-governmental organisations (NGOs) and concerned citizens worked to create a sense of urgency to build a broad-based coalition of support for action against ozone depletion.^21^ In the USA, NGOs leveraged negotiations of the Clean Air Act and regulations by the Environmental Protection Agency and launched judicial actions to address government inaction.^21 37^ Images of the ‘ozone hole’ with visualisations of what the ozone layer would look like with and without international action^38^ motivated civil society to keep countries accountable to protocol obligations and eventually shifted consumer preferences away from purchasing ODS-containing products.^39^ This highly mobilised public advocacy is credited as a key factor in the protocol’s enduring success.

**Targets and enforcement mechanisms (institutions)**: Countries that signed the protocol by 1987 were bound to meet targets to freeze production within 2 years, reduce ODS emissions by 75% within 5 years and nearly completely phaseout use by 1996 or within 7 years of entry into force.^23^ To ensure compliance with this new institutional architecture, ODS reduction schedules for all non-Article 5 countries were based on 1986 country-level production and consumption volumes. These strict schedules might have dissuaded countries from adopting the protocol if not for Article 4 prohibiting the trade of ODS with non-parties, which relied on a new system for monitoring the import and export of new, used, recycled and reclaimed ODS. With the prospect of losing access to major economic markets, including the USA, Soviet Union and the European Community, non-signatories were limited to trading ODS products to a shrinking portion of the global economy.^23^ This led to a continual expansion of treaty signatories with a concurrent ratcheting up of treaty commitments.

**Independent external assessments (ideas)**: Article 6 established independent monitoring mechanisms through Scientific and Environmental Effects panels to evaluate new ideas and ensure independent accountability.^4^ These panels bring together hundreds of experts to review emerging findings and produce recommendations on strengthening the protocol by regulating new chemical products, revising schedules and ensuring country compliance.^40^ The scientific assessment panel, for example, informs signatories of atmospheric chemical concentration changes of ODS controlled under the protocol such as CFCs and halons, and non-controlled substances, such as methylchloride and very short-lived substances, which have fewer effects on the ozone layer.^9^

**Lessons for AMR**: Building an informed and mobilised advocacy coalition to catalyse collective action and adopting global goals and country-specific targets that can be monitored will be key to spurring global action on AMR.^41^ However, despite mounting scientific evidence on AMR health risks, there arguably is not enough global public pressure to hold governments accountable for action.^42^ The Montreal Protocol represents an example of the role public advocacy can play to influence the design and implementation of treaties and highlights the importance of having independent expert assessments. As proposed by the Interagency Coordination Group in 2019, the establishment of an Independent Panel on Evidence for Action (IPEA) against AMR will allow independent assessments to provide countries with regular reports on the science of AMR, its impacts, and future risks, and to recommend options for adaptation and mitigation.^43^ However, even though an Advisory Group was set up in 2020 to draft terms of reference of the IPEA, the panel has not yet been established.

## Limits to learning from the Montreal Protocol

It is important to acknowledge the limitations to applying the Montreal Protocol model to AMR governance, including stronger issue complexity and stakeholder diversity, differing market and incentives structures, and higher resource requirements in the case of AMR, as well as differing impact timelines.^44 45^ For example, the progressive timeline and consumption thresholds used in the protocol may need to be modified. Unlike the impact of ODS on ozone depletion, the emergence of resistant microbes can occur rapidly, with immediate consequences for health and well-being of affected populations. Additionally, the scale of funds required to manage AMR may be orders of magnitude greater than what the MLF delivered. The total global cost of implementing containment measures and NAPs is estimated to be between US$4 billion and US$9 billion per year.^8^ However, by the end of 2022, only approximately US$26.6 million had been committed and US$19 million deposited to the Multi-Partner Trust Fund on AMR.^46 47^ Underinvestment in AMR to date represents a missed opportunity as investments in AMR containment are expected to yield high annual rates of return.^8^

Moreover, the commercial interests tied to antimicrobial use, like the pharmaceutical industry’s role in antibiotic development and distribution, differ from those of CFC conglomerates.^8 12 48^ Therefore, an effective governance mechanism for AMR must navigate a more adaptive approach to working with these commercial interests and a vast set of relevant AMR stakeholders, while ensuring equitable access and greater antimicrobial stewardship. Also, ethical concerns around equitable access to antibiotics and the impact of trade restrictions on public health are more pronounced in the case of AMR. Using enforcement mechanisms such as trade restrictions to prohibit non-party countries from receiving antibiotics for human use would be unethical, but such mechanisms could be leveraged to regulate the unsustainable use of antibiotics in agriculture and animal husbandry. Even with such enforcement, improving current prescription habits among clinicians, changing patients’ understanding of antibiotics use and regulating consumption in agricultural and veterinary practices will require broader public engagement to incentivise behavioural changes, as compared with the focus on industrial practices in the Montreal Protocol.

Finally, other environmental treaties may offer complementary strategies that should be considered for the global governance of AMR. Nonetheless, the Montreal Protocol’s historical and measurable success, universal adoption and relevance to global health make it a natural case from which to draw evidence-informed insights. Future research should consider conducting comparative analyses of multiple treaties to provide a more comprehensive framework for global governance strategies in addressing AMR.

## Conclusion

Like ozone depletion, failure to take global action on AMR threatens millions of lives and imposes substantial development costs if adequate control measures are not implemented.^8 24^ Moreover, AMR and ozone depletion are both complex challenges that—while engaging different sets of stakeholders—share a need to engage with diverse industry and regulatory actors due to their global scope, cross-sector impact, long-term consequences, reliance on scientific research, the importance of preventive measures, regulatory policies and public awareness all accentuate their interconnectedness. Overcoming current AMR challenges, such as uneven antimicrobial stewardship, and inadequate political will to catalyse research and investment in the discovery of new antimicrobials, requires sustainable MLF, multisectoral coordination and strong global advocacy. We highlight equity, flexibility and accountability as key components of the Montreal Protocol’s success in bringing together a range of actors across sectors with competing interests to reduce global ODS emissions and ultimately restore the ozone layer’s protective effects for human, animal and environmental health. With 40 years of hindsight, similar intersectoral challenges and measurable evidence of success, it would be a mistake to overlook the hard-won lessons of this global success story in developing a truly effective, adaptive and equitable global agreement to manage the global antimicrobial commons.

## Data Availability

There are no data in this work.
